# Age‐specific melanoma risk associated with melanocytic nevi: a pooled analysis from the M‐SKIP project

**DOI:** 10.1111/ddg.15992

**Published:** 2026-03-09

**Authors:** Giulia Doi, Aurora Gaeta, Simone Ribero, Nelleke Gruis, Julia Newton‐Bishop, David Polsky, DeAnn Lazovich, Paola Ghiorzo, Gloria Ribas, Chiara Menin, Alexander J. Stratigos, Gabriella Guida, Susana Puig, Maria Concetta Fargnoli, Peter A. Kanetsky, Paola Queirolo, Vincenzo Bagnardi, Veronique Bataille, Sara Raimondi, Sara Gandini, M‐SKIP study group

**Affiliations:** ^1^ Molecular and Pharmaco‐Epidemiology Unit Department of Experimental Oncology IEO European Institute of Oncology IRCCS Milan Italy; ^2^ Department of Statistics and Quantitative Methods University of Milan‐Bicocca, Italy; ^3^ Department of Medical Sciences Dermatologic Clinic University of Turin Turin Italy; ^4^ Department of Dermatology Leiden University Medical Center Leiden The Netherlands; ^5^ Institute of Medical Research at St James's University of Leeds Leeds UK; ^6^ Ronald O. Perelman Department of Dermatology New York University Grossman School of Medicine NYU Langone Health New York USA; ^7^ Division of Epidemiology and Community Health University of Minnesota Minneapolis Minnesota USA; ^8^ Department of Internal Medicine and Medical Specialties University of Genoa Genoa Italy; ^9^ Cancer Genetics IRCCS Ospedale Policlinico San Martino Genoa Italy; ^10^ Dpdt. Oncologia medica y hematologia Fundación Investigación Clínico de Valencia Instituto de Investigación Sanitaria‐ INCLIVA Valencia Spain; ^11^ Diagnostic Immunology and Molecular Oncology Unit Veneto Institute of Oncology IOV‐IRCCS Padua Italy; ^12^ 1st Clinic of Dermatological and Venereal Diseases Medical School National and Kapodistrian University of Athens Andreas Sygros Hospital Athina Greece; ^13^ Department of Translational Biomedicine and Neuroscience (DiBraiN) University of Bari “A. Moro” Bari Italy; ^14^ Melanoma Unit Dermatology Department Hospital Clinic Barcelona Universitat de Barcelona Institut d'Investigacions Biomèdiques August Pi I Sunyer (IDIBAPS), Spain; ^15^ CIBER de Enfermedades Raras Barcelona Spain; ^16^ San Gallicano Dermatological Institute – IRCCS Rome Italy; ^17^ Department of Cancer Epidemiology H. Lee Moffitt Cancer Center and Research Institute Tampa Florida USA; ^18^ Melanoma Sarcoma and Rare Tumors Oncology Department IEO European Institute of Oncology IRCCS Milan Italy; ^19^ Twin Unit and genetic epidemiology department King's College London London UK; ^20^ Current Affiliation: Recordati SpA Milan Italy

**Keywords:** Age, melanoma, MC1R, nevi, primary and secondary prevention

## Abstract

**Objective:**

This study investigates the association of melanoma risk with total‐body nevus count and the presence of atypical nevi in younger (< 40 years) and older (> 60 years) individuals.

**Methods:**

A pooled analysis was conducted within the M‐SKIP project, based on multiple melanoma case‐control studies. Associations were assessed through study‐specific odds ratios (ORs), adjusted for potential confounders.

**Results:**

Nine case‐control studies were included, analyzing common nevi (2,674 melanoma cases/2,343 controls) and atypical nevi (2,459 cases/1,740 controls). High common nevus count was associated with increased melanoma risk in both younger (summary [s]OR = 2.56; 95% CI 1.64‐4.01; I^2^ = 31%) and older individuals (sOR = 2.06; 95% CI 1.06–4.00; I^2^ = 67%), with no significant age‐group difference (p = 0.57). In contrast, younger individuals exhibited a markedly higher risk of melanoma associated with the presence of at least one atypical nevus (sOR = 4.84, 95% CI 2.18–10.76; I^2^ = 65%) compared to older individuals (sOR = 1.71 95%CI 1.07–2.74; I^2^ = 39%), with a p value for the difference of 0.02.

**Conclusions:**

These findings suggest age‐specific differences in melanoma risk associated with nevus burden. Specifically, atypical nevi are a stronger risk factor in younger individuals. This could have significant implications for prevention strategies and clinical management, in particular for better identification of high‐risk groups for targeted secondary prevention efforts.

## INTRODUCTION

Melanoma, characterized by the uncontrolled growth of melanocytes, is one of the most malignant forms of skin cancer,^1^ and among the most common cancers in younger individuals.^2^ Although less common than other types of skin cancer, its incidence is rising globally, raising serious public health concerns.^3^ The risk of melanoma increases with age; however, incidence rates vary by age and gender. While men are generally more likely to develop melanoma and have a higher risk of mortality, women under 50 are nearly twice as likely as men to be diagnosed with the disease.^4^ Understanding these demographic patterns is essential for developing targeted prevention and management strategies.

The risk of developing cutaneous melanoma increases with increasing number of common melanocytic nevi.^3^ Total body nevus count (TBNC) is one of the strongest risk factors for melanoma, with much higher relative risks than environmental exposure.^5^ Sunlight may be involved in nevogenesis, but nevi are mostly under genetic control, as shown by family and twin studies.^6^ Cross‐sectional studies, to date, have shown a lower prevalence of nevi in older age groups, yet they do not address the natural history of nevi which can only be loosely inferred from their findings.^7^, ^8^, ^9^ Understanding melanoma and nevus biology could help tailor secondary prevention strategies.

Based on previous cross‐sectional studies, it has been speculated that nevi typically evolve after the fourth decade of life in Caucasian populations and are rarer in the elderly. However, individuals susceptible to melanoma often have a larger number of common and atypical nevi, which persist until middle age or beyond.^3^ Most case‐control studies only offer cross‐sectional data, and thus it is not easy to accurately predict the behavior of nevi throughout a lifetime. Most longitudinal studies have been conducted in small high‐risk groups such as patients with melanoma, those with a family history of melanoma, or those with atypical nevi,^10^, ^11^, ^12^ and findings are unlikely to apply to the general population. Few longitudinal studies have examined whether nevus counts decrease with age and conflicting results have been reported.^10^, ^11^, ^12^

In this study, we aimed to analyze the relationship between nevi and melanoma according to age through a pooled analysis in a large sample size.

## MATERIALS AND METHODS

### Data

The data were derived from the M‐SKIP project, an international partnership on the melanocortin‐1 receptor gene (MC1R), skin cancer risk, and phenotypic characteristics.^13^ The authors of M‐SKIP provided information on the country, study design, source of controls, application of case‐control matching, phenotypic and genotyping methodology, and type of blindness. Variables such as age, gender, ethnicity, BMI, smoking status, sun exposure (intermittent and continuous/chronic), family history of skin cancer, melanoma body site, melanoma histology, Breslow thickness, hair and eye color, skin type, freckles, solar lentigines and number of common and atypical nevi were available. The M‐SKIP dataset included 39 research studies, but only 9 case‐control studies that provided information on age, sporadic cutaneous melanoma, and total body counts of common and/or atypical nevi were included. The continuous variable indicating the Total Number of Common Nevi (TNCN) was divided into categories. Where the TNCN was reported in categories, the median between the upper and lower counts of the class was taken. Studies with a number of common nevi equal to zero or missing were excluded. Furthermore, familial studies or studies with controls being subjects with an increased risk of skin cancer were not included. Additional details are presented in the Supplementary material.

### Analysis

**FIGURE 1:**
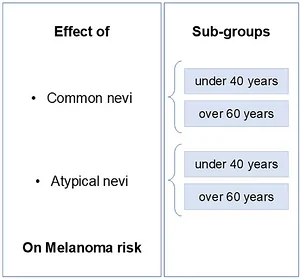
Subgroup analysis scheme.

Two independent analyses were performed (Figure 1): one regarding the impact of the total number of common moles on risk of melanoma, and the second regarding the association of at least one atypical nevus with melanoma risk. According to the IARC protocol for identifying and recording nevi in epidemiological studies,^14^ the definition of an atypical nevus is a mole with a macular component in at least one area, with at least three of the following features: size > 5 mm diameter, ill‐defined or blurred borders, irregular margin resulting in an unusual shape, varying shades of color (according to Fitzpatrick classification^15^), flat and bumpy components. Given the substantial diversity in the number of nevi in different age groups, we divided the sample into three subgroups: under 40 years,^16^ between 40 and 60 years, and over 60 years.^17^ Analyses were conducted comparing the two extreme age groups (< 40 years vs > 60 years). The intermediate age group (40 ≤ age ≤ 60) was excluded to allow assessment of an age‐specific effect.

### Pooled estimate

All the data collected were used for pooled analysis.^18^ The count of common nevi was transformed into a binary variable, categorized as above and below the study‐specific median count of nevi in the control group. The atypical nevi variable was defined as the presence of at least one nevus vs. the absence thereof. A logistic regression model was fitted to assess melanoma risk in each study, and odds ratios (ORs) with 95% confidence intervals (95% CIs) were reported. A multivariable model was fitted for each study, adjusting for any significant covariates. Where available, the following covariates were included: common/atypical nevi (for the two independent analyses), gender, intermittent and continuous sun exposure, phenotypic characteristics – such as hair and eyes color, skin type, family history of melanoma, and variants in the *MC1R* gene – considering the presence or absence of at least one variant of the gene. All the study‐specific ORs were combined into a pooled estimate with random‐effect model, which consists of an average of the single estimates weighted by their inverse marginal variances.^19^ Results were reported through forest plots.

Between‐study heterogeneity was investigated with the I^2^ statistic, which quantifies the proportion of total variability across studies attributable to heterogeneity rather than by chance. Meta‐regressions and subgroup analyses were performed, assessing the role of the characteristics of the study population and the methodological features of each study. In addition, meta‐regressions were used to investigate differences between younger and older subjects in the overall analysis. Sensitivity analyses were performed, also re‐including in the analysis studies with subjects at higher risk of melanoma, who had been excluded due to the inclusion criteria.

## RESULTS

The characteristics of the studies included are reported in Table 1: eight case‐control studies^20^, ^21^, ^22^, ^23^, ^24^, ^25^, ^26^, ^27^, ^28^, ^29^, ^30^, ^31^, ^32^, ^33^, ^34^, ^35^, ^36^, ^37^ were included in the common nevi analysis, involving 2,674 cases of cutaneous melanoma and 2,343 controls. The subgroup related to younger individuals had 605 melanoma cases and 433 controls, while the elderly dataset contained 533 cases of cutaneous melanoma and 615 controls. Polsky's study^36^ did not involve elderly people, and it was excluded in the analysis related to the older subgroup. Most of the studies were carried out in Europe, mainly in Italy, and for each case‐control study, the genotyping analyses were performed at a dedicated single center. The source of controls varied from study to study. The genotype was assessed by sequencing analysis, while phenotypical factors and nevi count were determined by examination of an expert or by self‐administered questionnaires.

**TABLE 1:** Characteristics of the studies included in the analysis.

First author	Year	Country	Cases/controls (N)	Age (mean – min/max)	Common nevi (median – IQR)	Source of controls	Assessment of number of nevi	Assessment of phenotype	Included in common nevi analysis	Included in atypical nevi analysis
Gruis^20^, ^21^, ^22^	2001	The Netherlands	114/343	55.49 (24.09–79.79)	14 (5–33)	Hospital	Examination by an expert	Examination by an expert	x	x
Ribas^23^	2007	Spain	102/155	51.50 (22–87)	12 (12–37)	Population	Self‐administered questionnaire or examination by an expert	Self‐administered questionnaire or examination by an expert	x	
Fargnoli^24^, ^25^, ^26^, ^27^	2008	Italy	155/162	49.34 (17–80)	30 (5–30)	Hospital	Examination by an expert	Examination by an expert	x	x
Bishop^28^	2009	UK	943/485	53.41 (17–86)	28 (13–60)	Hospital	Examination by an expert	Self‐administered questionnaire	x	x
Stratigos^29^, ^30^, ^31^	2009	Greece	73/120	46.02 (19–85)	5 (5–30)	Hospital	Examination by an expert	Interview by physician or examination by an expert (physician)	x	x
Kanetsky^44^, ^45^	2010	USA	713/245	49.25 (19.39–87.76)	/	Population	Examination by an expert	Self‐administered questionnaire		x
Ghiorzo^32^, ^33^, ^34^, ^35^	2012	Italy	330/216	54.73 (19‐85)	5 (5–30)	Healthy subjects	Examination by an expert for cases and controls	Examination by an expert for cases and half of controls (others self‐administered questionnaire)	x	x
Polsky^36^	2014	USA	875/765	46.36 (25–59)	45 (15–45)	Population	Self‐administered questionnaire	Self‐administered questionnaire	x	
Guida^37^	2015	Italy	82/97	52.59 (12–89)	25 (25–64)	Hospital	Examination by an expert	Examination by an expert	x	x
***Studies excluded from the main analysis but included in a sensitivity analysis***										
Menin^38^	2011	Italy	116/168	42.29 (15.64–82.52)	30 (5–30)	Healthy subjects	Examination by an expert for cases; self‐administered questionnaire for controls	Examination by an expert	x	x
Puig^39^, ^40^, ^41^, ^42^, ^43^	2013	Spain	278/113	51.06 (13.65–93.56)	25 (25–75)	High risk melanoma patients	Examination by an expert	Self‐administered questionnaire or examination by an expert	x	x

For each study, the table reports the following information: first author, publication year, country, number of melanoma cases and controls, mean age with minimum and maximum values, median and interquartile range (IQR) of common nevi, source of controls, methods for assessing number of nevi and phenotypic characteristics, and whether the study was included in the analyses of common and/or atypical nevi.

Subgroup analyses exploring between‐study heterogeneity are presented in the online supplementary Tables S1 and S2.

Raw and adjusted ORs for each study, considering a set of significant confounding variables, are presented in the online supplementary Tables S3a (younger group) and S3b (older group).

**FIGURE 2:**
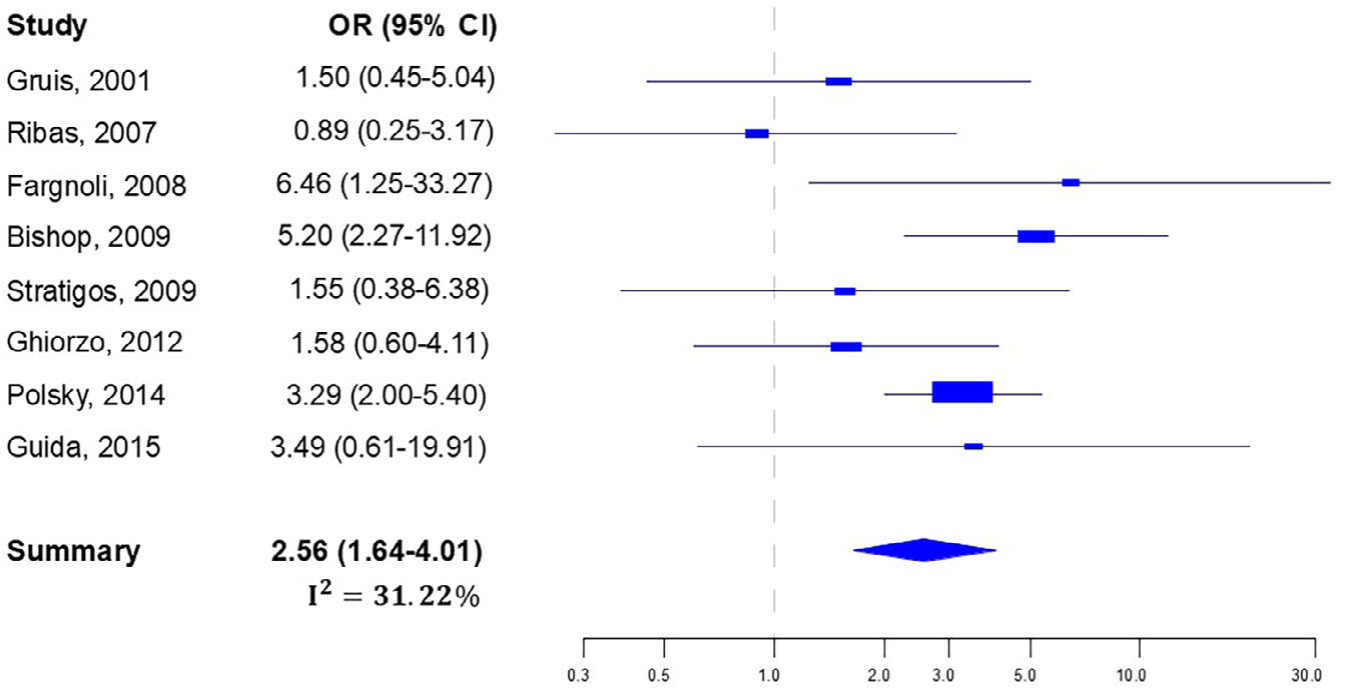
Forest plot of adjusted ORs for the association between common nevi and melanoma risk in the younger age group, comparing individuals with a number of common nevi below vs above the study‐specific median in the control group.

The summary OR (sOR) for the association between the presence of many common nevi and melanoma in younger subjects (< 40 years) was positive and significant: sOR = 2.56, 95% CI (1.64–4.01), with low between‐study heterogeneity 31.22% (Figure 2).

**FIGURE 3:**
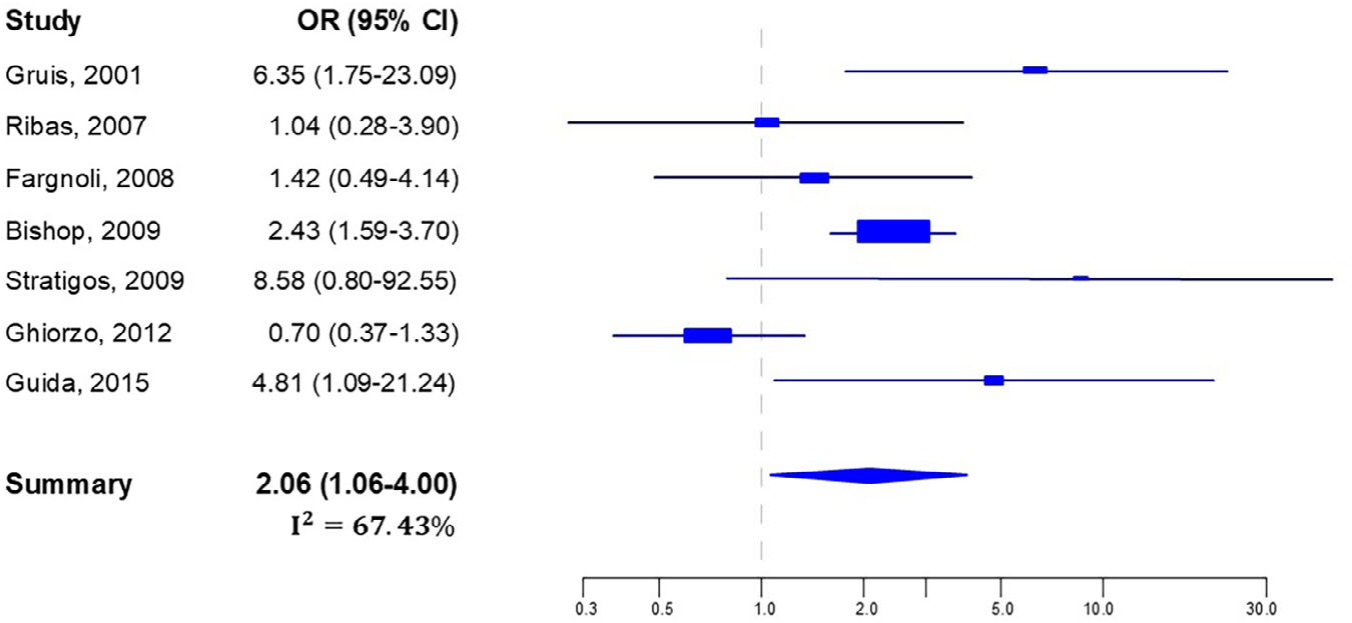
Forest plot of adjusted ORs for the association between common nevi and melanoma risk in the older age group, comparing individuals with a number of common nevi below vs above the study‐specific median in the control group.

For the older subgroup, as shown in Figure 3, the sOR = 2.06 (95% CI 1.06–4.00) indicated a significant doubling in the risk of melanoma for those with a high number of common nevi; however, a strong heterogeneity was found (I^2^ = 67.43%). Excluding Ghiorzo's data,^32^, ^33^, ^34^, ^35^ the value of I^2^ was 0 and the summary estimate became sOR = 2.48 (95% CI 1.75–3.50, Figure S1).

A meta‐regression analysis on the overall sample, which sought to investigate differences between the two groups (young vs. elderly), showed that there was no significant difference by age group for the risk of melanoma associated with common nevi (p = 0.57).

We subsequently carried out a sensitivity analysis, excluding the studies by Ribas and Stratigos,^23^, ^29^, ^30^, ^31^ as they presented an unexpected equal median number of common nevi for younger and elderly people in the control group.

After these exclusions, the summary OR for the association between the presence of many common nevi and melanoma in young people increased to sOR = 3.11 (95% CI 2.13–4.54, Figure S2), with a decreased heterogeneity (I^2^ = 6.13%). However, for the older group, the sOR was no longer statistically significant: sOR = 2.09 (95% CI 0.96–4.53) (Figure S3). The heterogeneity was found to be 75.7%, but, after eliminating the Ghiorzo's study,^32^, ^33^, ^34^, ^35^ the heterogeneity became equal to 0, with a sOR of 2.57 (95% CI 1.78–3.69). Nevertheless, in the overall sample meta‐regression, the risk of melanoma associated with a high number of common nevi showed no significant changes in risk by age group (p = 0.34).

We then conducted a second sensitivity analysis, including the cohorts from Menin and Puig,^38^, ^39^, ^40^, ^41^, ^42^, ^43^ which had previously been excluded due to noncompliance with the study's inclusion criteria (supplementary material). The summary OR for the association between the presence of many common nevi and melanoma in younger people (< 40 years) was 1.68 (95% CI 0.89–3.16) (Figure S4), showing no statistically significant association between a high number of common nevi and the risk of melanoma (p = 0.11); the between‐study heterogeneity was 69.59%. For older people, the summary estimate indicated a two‐fold increased risk: sOR = 2.00 (95% CI 1.09–3.68; I^2^ = 61.38%) (Figure S5).

Lastly, since the control groups varied across the different studies, we conducted a subgroup analysis, dividing the studies into those with hospitalized controls and those with general population controls or otherwise healthy subjects. We found that among younger individuals, there was no significant difference between the two groups of controls (p = 0.38). However, for older individuals, studies with hospitalized controls showed a significant sOR of 2.65 (95% CI 1.83–3.85), while studies with general population controls showed a non‐significant sOR of 0.76 (95% CI 0.42–1.35). In this case, the effect of study design on the association between the number of nevi and the risk of developing melanoma was statistically significant (p < 0.001).

### Atypical nevi

For atypical nevi, seven studies^20^, ^21^, ^22^, ^24^, ^25^, ^26^, ^27^, ^28^, ^29^, ^30^, ^31^, ^32^, ^33^, ^34^, ^35^, ^37^, ^44^, ^45^ were selected with 2,459 cases and 1,740 controls: 602/687 cases and 297/674 controls in the younger (< 40 years) and elderly people (above 60 years), respectively (Table 1).

**FIGURE 4:**
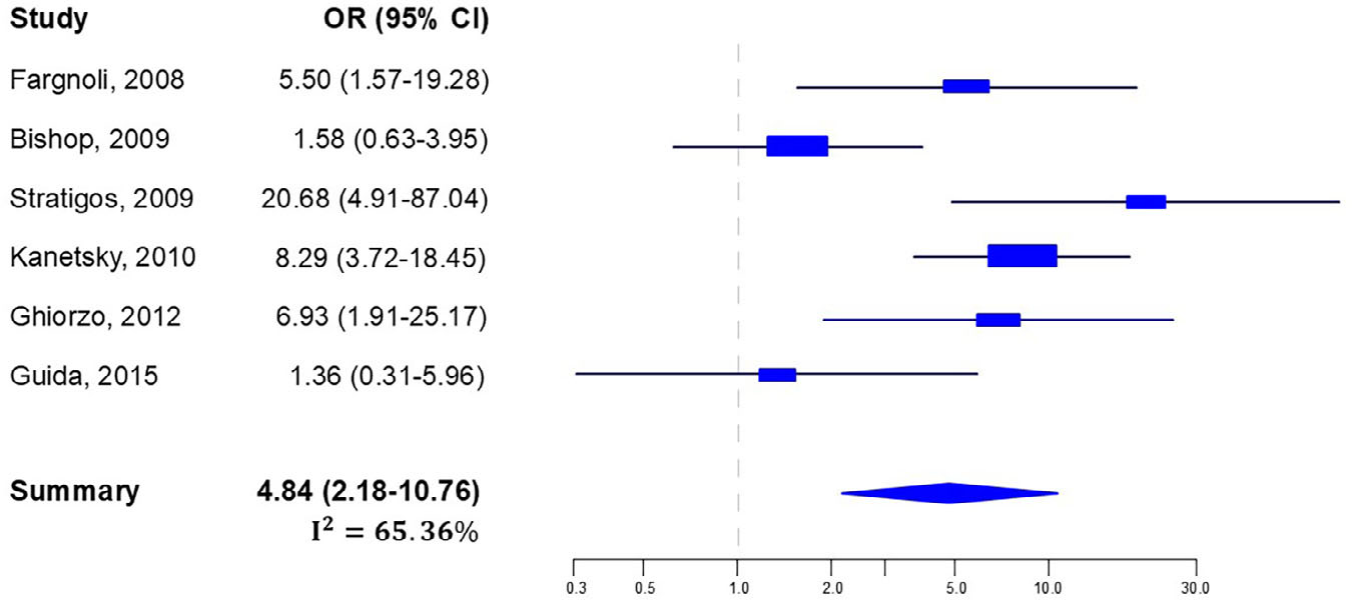
Forest plot of adjusted ORs for the association between atypical nevi and melanoma risk in the younger age group, comparing presence vs absence of atypical nevi.

In younger subjects, the sOR = 4.84 (95% CI 2.18–10.76) for the presence of at least one atypical nevus was significant, but with a high between‐study heterogeneity (I^2^ = 65.36%, Figure 4). One study, Bishop's,^28^ was mainly responsible for the heterogeneity. After this study was excluded, the association between the presence of any atypical nevus and the risk of melanoma remained significant: OR = 6.48 (95% CI 3.21–13.09) with an I^2^ of 40.56% (Figure S6).

Online supplementary Tables S4a (younger group) and S4b (older group) list the raw and adjusted ORs for each study, accounting for a set of significant confounding variables.

Including the Menin and Puig,^38^, ^39^, ^40^, ^41^, ^42^, ^43^ studies in the analysis also gave a similar total estimate of sOR = 5.71 (95% CI 2.21–14.72), but the heterogeneity increased significantly (I^2^ = 81.0%) (Figure S7).

**FIGURE 5:**
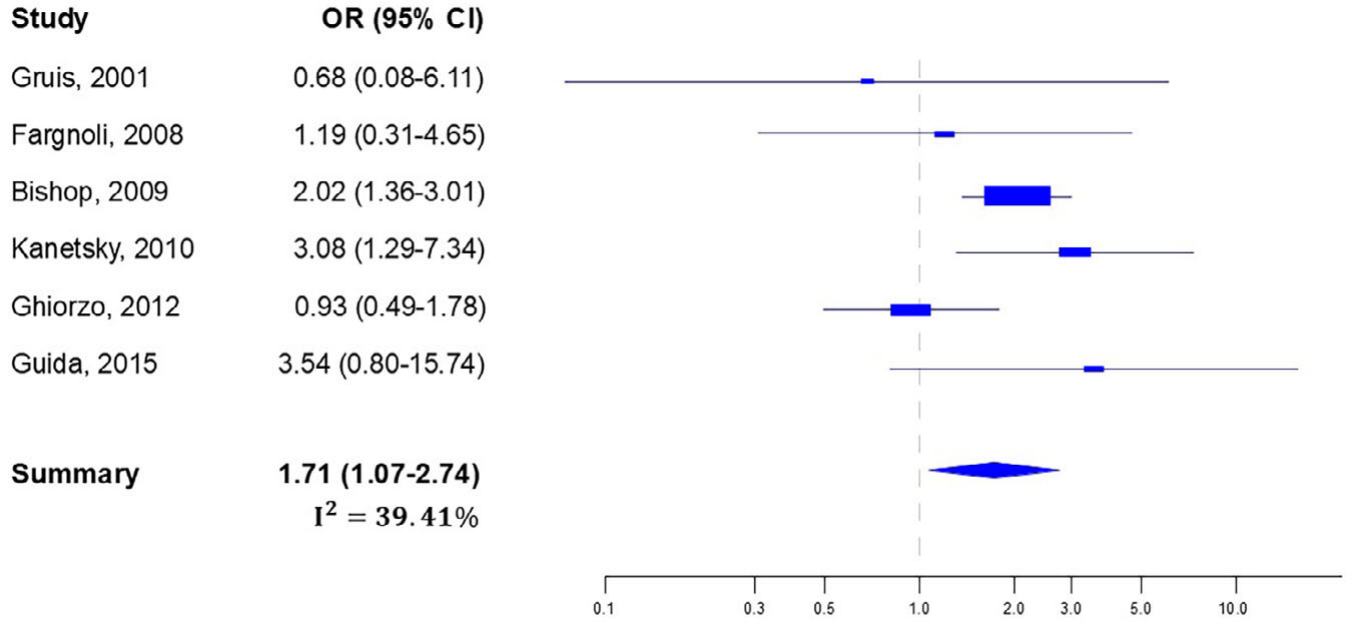
Forest plot of adjusted ORs for the association between atypical nevi and melanoma risk in the older age group, comparing presence vs absence of atypical nevi.

For older subjects, the risk of developing melanoma associated with one or more atypical nevi was much lower than for the younger age group (OR = 1.71; 95% CI 1.07–2.74) with a low heterogeneity (I^2^ = 39.41%) (Figure 5). Without Ghiorzo's study,^32^, ^33^, ^34^, ^35^ the heterogeneity was 0, with a value of the estimate OR equal to 2.09 (95% CI 1.49–2.93).

In the overall analysis, the variation in risk of melanoma in relation to any atypical nevi between the younger and older groups was statistically significant (p = 0.02).

## DISCUSSION

Melanoma risk is significantly influenced by nevi characteristics, with both the number and types of nevi acting as independent risk factors. A strong dose‐dependent relationship exists between total body nevus count and melanoma risk,^5^ with more than eleven moles on one arm serving as a predictor of high TBNC.^46^ While the likelihood of a single nevus transforming into melanoma is low, having multiple common and atypical nevi serves as a marker of higher melanoma susceptibility.^47^

Understanding melanoma risk across different age groups is crucial, as melanoma disproportionately affects younger individuals compared to many other cancers and identifying high‐risk groups may support more effective targeted secondary prevention efforts.

The evolution of nevi over a person's lifetime provides insights into melanocyte senescence.^48^ Interestingly, individuals with a high number of nevi tend to have a better prognosis for melanoma, including improved survival, possibly due to reduced cellular senescence in both melanocytes and immune cells.^49^

Nevi are growth‐arrested, clonal neoplasms of melanocytes driven by mutations in the mitogen‐activated protein kinase (MAPK) pathway, most commonly the activating BRAF V600E mutation.^50^ Nevi count peaks in the fourth decade due to a combination of reduced new nevus formation and regression of existing nevi.^48^ Dermoscopy studies show that nevi follow a typical life cycle: in childhood, they appear globular, transitioning to reticular patterns in adulthood, and eventually becoming compound or dermal before disappearing in older adults, particularly in non‐head and neck regions.^51^, ^52^, ^53^, ^54^

The clinical regression of nevi is not well understood but is known to involve immune signals and genetic factors such as BRAF, CDKN2A, and telomere‐related genes.^55^ Telomere length is related to nevus count, with individuals carrying homozygous loss‐of‐function mutations in P16 (CDKN2A‐null) sometimes developing numerous atypical nevi that persist with age and increase susceptibility to melanoma and other cancers.^56^

A systematic review^50^ of 708 studies found only two population‐based longitudinal studies meeting inclusion criteria for nevus count in adults with a baseline and at least one follow‐up nevus count.^57^, ^58^ Ribero et al. conducted a 15‐year longitudinal study showing that TBNC decreases with age, according to cross‐sectional data.^16^ However, longitudinal data suggested a slight increase in TBNC in older individuals, possibly due to misclassification of solar lentigines and seborrheic keratoses as nevi. The decline in TBNC observed in cross‐sectional studies may be partially explained by a cohort effect, as suggested by Plasmeijer et al.^50^

Building on these previous findings, our study aimed to further investigate the relationship between nevus count and melanoma risk across different age groups. Our results showed that, when comparing younger and older individuals, a high number of common nevi was not significantly associated with an increased melanoma risk compared to a low number (p = 0.57). However, the melanoma risk associated with a high number of common nevi was found to be significant in both groups, with OR of 2.56 and 2.06 in younger and older subjects, respectively. Adjusting for atypical nevi in common nevi analysis did not change results (adjusted p‐value = 0.39); similarly, this holds true, albeit to a lesser extent, when analyzing atypical nevi. Although clinically mole count appears to decrease with age, longitudinal studies and meta‐analyses have failed to pinpoint the exact moment in life when this occurs, likely explaining lack of a significant difference in melanoma risk across age groups.

Regarding atypical moles, melanoma risk was higher in younger individuals compared to older ones (sOR = 4.84 vs. 1.71), thus indicating that the risk of developing a melanoma based on the presence of one atypical mole compared to none is much higher in those below 40 years than in those above 60 years (p value = 0.02). This does not imply that a single atypical mole is highly likely to become melanoma; however, the presence of atypical nevi is a stronger predictor of melanoma risk in younger individuals.

The predictive value of atypical nevi declines in older individuals, possibly due to differences in melanoma pathways. According to Whiteman's two‐pathway model,^59^ melanoma can arise through distinct pathways: individuals with numerous nevi are more likely to develop nevus‐associated melanomas earlier in life with less cumulative sun exposure; conversely, older individuals are more likely to develop de novo melanomas after continuous sun exposure, which reduces the relative importance of nevus count as a risk factor in this population.

Screening practices may also have influenced these trends. Historically, atypical nevi in elderly patients were often excised without dermoscopic evaluation to prevent malignant transformation, potentially altering the perceived risk by removing them before they could be included in clinical studies. However, this hypothesis could not be confirmed due to a lack of retrospective scar count data in cases and controls.

### Strengths and limitations

This study consisted of a pooled analysis.^18^ Data from over 30 studies in the M‐SKIP dataset were collected,^13^ allowing for robust subgroup analyses by age and nevus type, and facilitating the investigation of rare exposures and the formulation of new hypotheses. By integrating data from multiple studies, statistical power was enhanced, allowing for a more precise estimation of melanoma risk. Researchers were able to highlight and quantify age‐related differences while minimizing total heterogeneity. Additionally, the standardization of variables across different studies allowed for adjustments based on multiple confounders such as sex, sun exposure, and *MC1R* mutations, further improving the reliability of the findings.

However, the study's retrospective design posed challenges, as original studies had different research objectives. Data collection methods varied across studies, requiring standardization to create a coherent dataset, and leading to OR adjustments for different sets of confounders across studies. Moreover, differences in nevus distribution across studies prevented definition of a unified median, so the median nevus count in each control group was used as a reference; this variability may have influenced the results, although efforts were made to standardize data analysis.

## CONCLUSIONS

Overall, the study provides valuable insights into age‐related variations in melanoma risk based on nevus count and atypical nevi. These findings underscore the importance of age‐specific risk assessment and highlight the need to integrate genetic predisposition and improved longitudinal monitoring of nevus evolution to support more targeted secondary prevention strategies.

## CONFLICT OF INTEREST STATEMENT

None.

## Supporting information



Supplementary Information
